# Cheyne-Stokes Breathing as a Predictive Indicator of Heart Failure in Patients With Obstructive Sleep Apnea; A Retrospective Case Control Study Using Continuous Positive Airway Pressure Remote Monitoring Data

**DOI:** 10.3389/fcvm.2022.790331

**Published:** 2022-02-07

**Authors:** Kimimasa Saito, Yoko Takamatsu

**Affiliations:** Saito Naika Kokyukika, Mie Sleep Clinic, Ise-shi, Japan

**Keywords:** continuous positive airway pressure, obstructive sleep apnea, Cheyne-stokes breathing, remote monitoring, retrospective case control study, heart failure with preserved ejection function, predictive indicator, cycle length

## Abstract

**Objective:**

We conducted a retrospective case control study to examine whether remote monitoring of Cheyne-Stokes breathing (CSB) was useful for predicting the onset of heart failure (HF) in patients with obstructive sleep apnea (OSA) on continuous positive airway pressure (CPAP).

**Methods:**

Among patients with OSA treated at our hospital, 33 patients with HF that occurred between July 2014 and May 2021 [11 patients with acute HF (AHF); 22 patients with chronic HF (CHF) exacerbation] were included in the HF group. Of the 618 stable patients, 149 patients with a 30-days average CSB rate (CSB%) ≧1% were included in the non-HF control group. The chronologic change of CSB% were compared among the AHF, CHF and Control groups. Furthermore, of the 149 patients in the non-HF control group, 44 patients were matched for CSB%, body mass index, and sex in a ratio of 1:2 to 22 patients with CHF. The average cycle length (CL) of CSB was compared among three groups: CHF in stable period (CHF-stable group), CHF in exacerbation period (CHF-exacerbation group), and control group. In addition, according to the status of HF, receiver operating characteristic (ROC) curves were generated to determine the optimal cut-off points for variation of CSB% and CL.

**Results:**

Chronological change in CSB% among the three groups was significantly different. Standard deviation of CSB% (SD CSB%) before onset HF was significantly higher in both the AHF and CHF groups than in the control group. The CL of CSB was significantly longer in the CHF group than in the control group and was longer during the exacerbation period than during the stable period. The optimal cut-off value of CL that could differentiate patients with and without the onset of HF was 68.9 s.

**Conclusion:**

The HF group demonstrated greater CSB variations and longer CL than the non-HF control group. Furthermore, the CL was longer during the exacerbation period of HF even in the same patient. These results suggest that remote monitoring of CPAP device data for CSB variations and CL might allow early prediction of the onset and exacerbation of HF.

## Introduction

The respiratory status has been reported to reflect hemodynamics. In a prospective study on patients with heart failure (HF) and New York Heart Association functional class II to IV, Kumagai et al. quantitatively measured diurnal respiratory instability in patients during bed rest while awake, and reported that the diurnal respiratory instability was an independent predictive indicator of outcomes such as unscheduled hospitalization due to exacerbation of HF (1). During sleep, sleep-disordered breathing that occurs in association with HF, such as obstructive sleep apnea (OSA) and central sleep apnea (CSA) with Cheyne-Stokes breaking (CSB) (CSA-CSB), have been targeted for treatment. Since publication of the results of the Treatment of Predominant Central Sleep Apnea by Adaptive Servo Ventilation in Patients with Heart Failure (SAVE-HF) trial (2), and a study demonstrating that adaptive servo ventilation (ASV) does not alleviate nocturnal cardiovascular stress in patients with systolic heart failure and predominant CSA (3), as well as other studies, the importance of CSA-CSB as a treatment target, particularly in patients with impaired left ventricular function, has been reduced. On the other hand, CSA-CSB occurs in the acute phase of HF and often decreases or disappears with improvement in HF due to drugs or other treatments.

The technological advances in continuous positive airway pressure (CPAP) devices have enabled remote monitoring of various respiratory events during sleep in patients on CPAP (4). In real-world clinical practice, it is often observed that remote monitoring of CSB may allow much earlier detection of new onset as well as exacerbation of pathological conditions, especially cardiovascular disease, in OSA patients (5–7).

**Figure 1:**
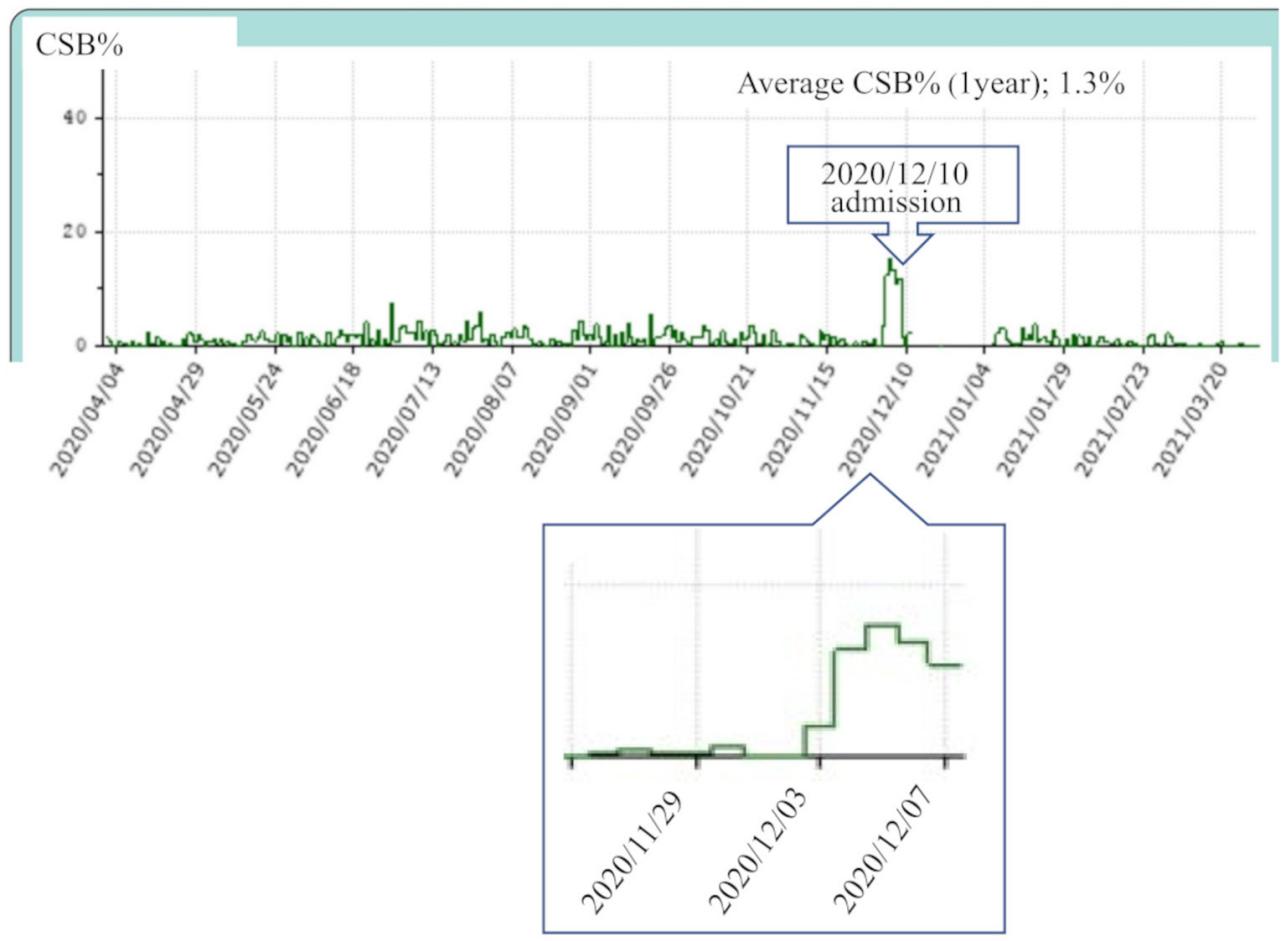
This patient visited our hospital for acute heart failure on December 10, 2020 and was admitted to the coronary care unit of a specialized hospital on the same day. Continuous positive airway pressure remote monitoring data were collected from April 2020 to March 2021. The average Cheyne-Stokes breathing rate (CSB%) for 1 year was 1.3%. The CSB% clearly increased from that around December 3, 1 week before the day of presentation.

Figure 1 shows the data collected at our hospital over 1 year through CPAP remote monitoring of a patient with OSA who developed acute HF. The data revealed a clear increase in CSB 8 days before the patient made an unscheduled visit to our hospital with the chief complaint of orthopnea and was transferred to a specialized hospital for admission to the intensive care unit (December 3, 2020).

In this retrospective case control study, we examined whether remote monitoring of CSB was useful for predicting the onset of HF in patients with OSA on CPAP.

## Methods

### Ethics Statement

This study was approved by the ethics committee of the Medical Corporation MSC (#20002) and written consent was obtained from all patients. This study is registered in the UMIN Clinical Trials Registry (UMIN000042555).

### Definition of Heart Failure

Clinical syndrome consisting of dyspnea, malaise, swelling and/or decreased exercise capacity due to the loss of compensation for cardiac pumping function due to structural and/or functional abnormalities of the heart (8).

#### Acute HF

*De novo* heart failure (patients with no history of hospitalization for heart failure).

#### Chronic HF

Patients with history of hospitalization for heart failure.

##### Definition of Exacerbation

Moderate limitation of physical activity. Physical findings such as edema. In addition, the BNP level and Chest X-ray or Echocardiography findings were used as reference for judgment (9).

CHF classified them into three groups according to left ventricular ejection fraction (LVEF): HF with reduced EF (HFrEF; EF < 40%), HF with mid-range EF (HFmrEF; 40% ≤ EF < 50%), and HF with preserved EF (HFpEF; 50% ≤ EF) (8).

### Subjects

#### HF Group

The HF group included patients treated with CPAP at Mie Sleep Clinic who developed HF between July 2014 and May 2021. We examined a total of 33 patients, including 11 patients with AHF (AHF group) and 22 patients with CHF (CHF group). When the same patient with CHF experienced repeated HF events, an event that occurred after an event-free period of 6 months was counted as one case of exacerbation. In all patients, HF was diagnosed by cardiologists at nearby core hospitals.

AHF was clinically classified according to the Clinical Scenario classification (10), and CHF was clinically classified according to the New York Heart Association functional classification (11).

#### Control Group

**Figure 2:**
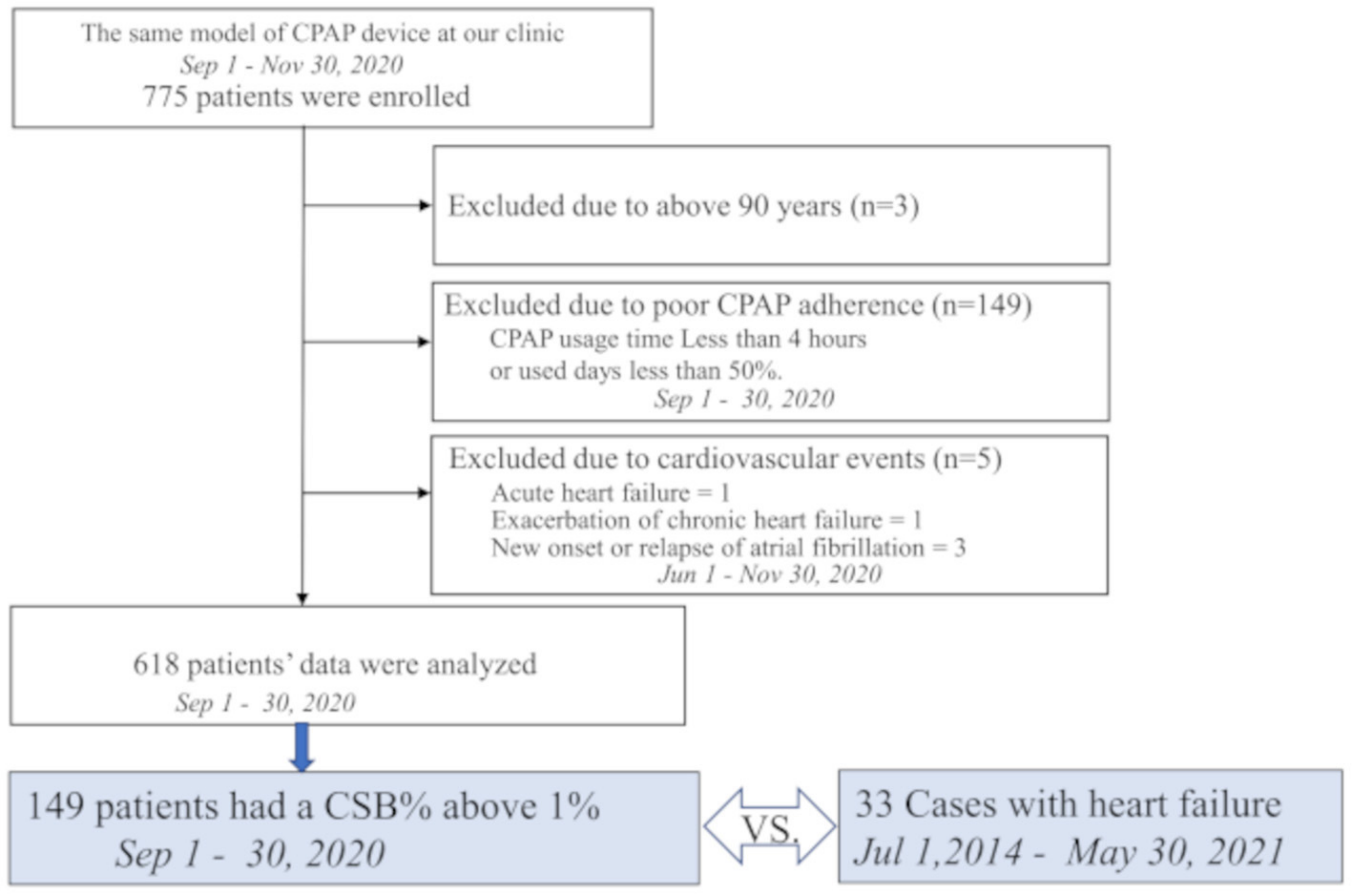
Study profile of 775 patients who were receiving continuous positive airway pressure (CPAP) using the same device model at our hospital on September 1, 2020. Among them, 149 patients had an average Cheyne-Stokes breathing rate (CSB%) of 1% or higher over a 1-month period lasting from September 1 to 30, 2020. These 149 patients were selected as the control group and compared with 33 patients who developed heart failure.

Previously we conducted a retrospective cohort study on 775 patients who had used the Dream Station Auto CPAP machine (Philips Respironics, Murrysville, PA) for at least 1 year at Mie Sleep Clinic as of September 1, 2020. All patients were diagnosed with OSA, with an apnea-hypopnea index (AHI) ≥20 by overnight polysomnography, or with sleep apnea syndrome, predominantly of the obstructive type, with AHI ≥40 by out-of-center sleep testing, and had been continuously treated with CPAP. Based on the data obtained in that study, we selected a control group of 149 patients with an average CSB rate (CSB%) of 1% or higher over 30 days from September 1, 2020 (Figure 2).

Exclusion criteria were as follows: age ≥90 years as of September 1, 2020; poor-adherence patients with the percentage of days of CPAP use <50% or an average CPAP use time <4 h; new onset of HF, worsening of CHF, or new onset or relapse of atrial fibrillation (Af), or other cardiovascular diseases in the 3 months before or after September 1, 2020 (i.e., between June 1, 2020 and November 30, 2020).

### Definitions of CSB% Detected by the CPAP Device

The American Academy of Sleep Medicine recommends scoring a respiratory event as CSB if both of the following criteria are met:

a. There are episodes of at least three consecutive central apneas and/or central hypopneas separated by a crescendo and decrescendo change in the breathing amplitude, with a cycle length of at least 40 s (typically 45–90 s).
b. There are five or more central apneas and/or central hypopneas per hour associated with a crescendo/decrescendo breathing pattern recorded over a minimum of 2 h of monitoring.

According to the algorithm of the CPAP device, the device detects periodic breathing that meets only criterion a. Thus, in this study, we defined CSB% as the percentage (%) of time exhibiting periodic breathing to the duration of the CPAP device use as recorded in the CPAP data.

### Study Design

**Figure 3:**
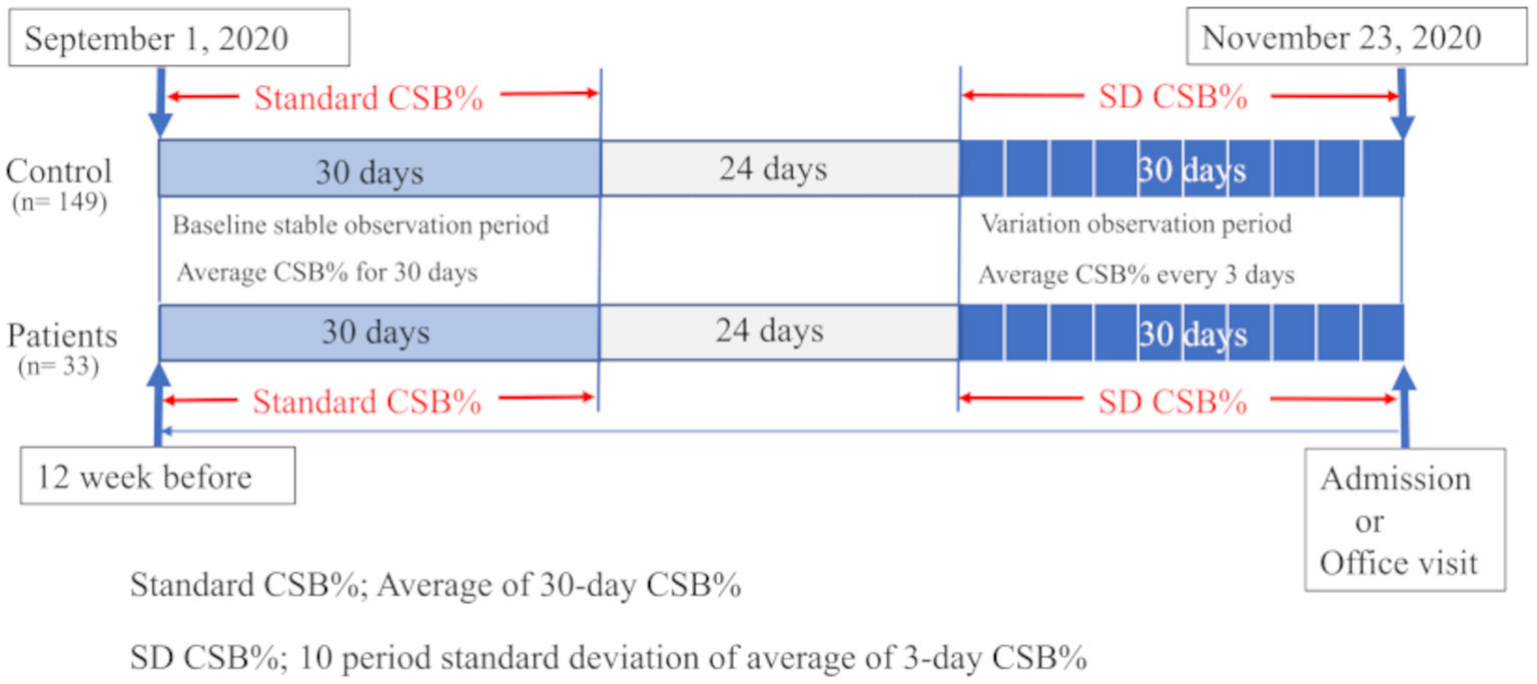
Study protocol is shown. In 33 patients with heart failure, the average Cheyne-Stokes breathing rate (CSB%) was calculated for a 30-day period starting 12 weeks before the day of the emergency visit or admission and regarded as the standard CSB%. In the 149 controls, the standard CSB% was calculated as the average CSB% from September 1 to 30, 2020. To evaluate the variations in Cheyne-Stokes breathing, the first 3-day period was considered to start 30 days before the base date in patients with heart failure. The variations in the average CSB% for 10 periods up to the base date were calculated as the standard deviation (SD) of CSB%. In the control group, the 3-day periods were set during a period from October 24 to November 23, 2020. The variations in the average CSB% for 10 periods were calculated as SD CSB%.

As shown in Figure 3, in the HF group, the base date was defined as the day when patients were admitted to hospital or visited our outpatient clinic for new onset of HF (AHF) or exacerbation of CHF. We assumed that they were in a stable period for 30 days starting 12 weeks before the base date. The average CSB% in the stable period was regarded as the standard CSB% of the HF group. In the control group, the standard CSB% was calculated as the average CSB% from September 1 to 30, 2020.

For CSB variations, the average CSB% was calculated for each period of 3 days. The variations in the average CSB% for a total of 30 days, or in other words, 10 periods, were evaluated by standard deviation (SD CSB%). In the HF group, the first 3-day period started 30 days before the base date. The standard deviations in the average CSB% over 10 periods up to the base date were calculated as SD CSB%. In the control group, the 3-day periods were set during a period from October 24 to November 23, 2020. The standard deviations in the average CSB% over 10 periods were calculated as SD CSB%.

We prepared a graph showing the chronological changes in the 3-day average CSB% over a total of 30 days, or in other words, 10 periods, in three groups consisting of the AHF, CHF, and control groups. We also compared the differences in the SD CSB% among the three groups. For the HF and control groups, a receiver operating characteristic (ROC) curve was generated to determine the optimal cut-off value of SD CSB%.

In addition, from the 149 patients in the control group, we selected 44 patients who were pair matched for sex, body mass index (BMI), and CSB% in a ratio of 1:2 to 22 patients in the CHF group. The cycle length (CL) of CSB was compared between these groups. The CL was calculated from the CPAP remote monitoring data obtained on any day with raw waveforms between September 1 and 30, 2020, in the control group and on any day with raw waveforms close to the base date and during the stable period (~12 weeks before the base date) in the CHF group. The CL (s) was calculated as an average of all intervals between peaks of measurable spindle-shaped respiratory waveforms during a period with CSB identified with the help of the analysis software.

The CL was compared among the three groups: CHF in the stable period (CHF-stable), CHF in the exacerbation period (CHF-exacerbation), and controls, and between the CHF-stable and CHF-exacerbation groups. For the CHF-stable and control groups, an ROC curve was generated to determine the optimal cut-off value of CL.

### Clinical Data Collection

The baseline characteristic data extracted from the Medical Corporation MSC Mie Sleep Clinic database, which contained data from October 1, 2007 to May 30, 2021, with the base point set as September 1, 2020. Echocardiography data for the control group were taken from the database performed between May 1, 2019 and November 30, 2020.

### Sleep Data Collection

The CPAP device used was the Dream Station Auto. The device periodically records the user's breathing signals and analyzes the data in near real time. It also records raw respiratory data at random. Analysis results are automatically uploaded to a central database. The data were analyzed online using the dedicated analysis system (EncoreAnywhere, ver. 2.44; Philips Respironics) and cited using the CareExporter software (version 1.11.1.0; Philips Respironics). The data cited were average apnea/hypopnea index (AHI), average central apnea (clear airway) index, average CSB%, percentage of days of CPAP use (%), and average CPAP use time (in days of use, min).

### Statistical Analysis

Data are presented as numbers (%) or means ± standard deviation (SD).

Two-group comparisons were performed using the Mann–Whitney U test for continuous variables, and Fisher's exac*t*–test for nominal variables. The Wilcoxon signed rank test was used to compare the CL between the stable and exacerbation periods of CHF. The two-way repeated measures ANOVA was used to compare the differences in mean chronological change among the three corresponding groups. Differences among the three groups were analyzed using the Kruskal–Wallis test, and multiple comparisons were performed using the Steel–Dwass test. To determine the optimal cut-off value of SD CSB%, an ROC curve was generated for the HF and control groups. To determine the optimal cut-off value of CL, an ROC curve was generated for the CHF-stable and control groups.

Probability values ≤ 0.05 were considered significant. All statistical analyses were performed using EZR Ver.1.52 (Saitama Medical Center, Jichi Medical University, Saitama, Japan), which is a graphical user interface for the R version 4.02 (The R Foundation for Statistical Computing, Vienna, Austria). Specifically, it is a modified version of the R commander designed to add statistical functions frequently used in biostatistics.

## Results

### Baseline Characteristics

Table 1 shows the baseline characteristics and various indices accumulated by the CPAP device during the stable period in the HF group consisting of 33 patients (11 with AHF and 22 with CHF), and control group consisting of 149 patients. Both groups predominantly consisted of men. Although the HF group tended to be older than the control group (76.2 ± 5.6 vs. 66.9 ± 12.5 years, *P* < 0.001), no significant difference was observed in the BMI (26.3 ± 3.8 vs. 27.9 ± 5.0, *P* = 0.055). Despite statistically significant differences in the number of CPAP usage days, adherence to CPAP was extremely favorable in both groups. The average usage years of CPAP did not significantly differ between the groups (8.8 ± 3.2 vs. 8.1 ± 3.9, *P* = 0.573). As for the sleep indices obtained by the CPAP device during the stable period, AHI was not significantly different (7.9 ± 6.5 vs. 6.3 ± 4.8, *P* = 0.210), whereas the standard CSB% was significantly higher in the HF group (8.4 ± 9.2 vs. 4.4 ± 6.1, *P* = 0.013). Regarding comorbidities, the comorbidity rates of Af and stage 3b or higher nephropathy were significantly higher in the HF group (78.8 vs. 30.2%, *P* < 0.001 and 27.3 vs. 8.1%, *P* < 0.001, respectively). No significant differences were observed in the QRS duration or comorbidity rate of hypertension between the two groups (105.8 ± 21.2 vs. 99.5 ± 15.1 ms, *P* = 0.154 and 72.7 vs. 63.8%, *P* = 0.420, respectively).

**Table 1:** Baseline characteristics and various indices from CPAP Device accumulated in stable period.

**Variables**	**AHF** **(*n* = 11)**	**CHF** **(*n* = 22)**	**Overall HF** **(*n* = 33)**	**Control** **(*n* = 149)**	***p*** **-value**
Male (%)	10 (90.9)	22 (100)	32 (97.0)	143 (96.0)	1
Age, year	74.5 ± 7.7	77.0 ± 4.2	76.2 ± 5.6	66.9 ± 12.5	***p*** **< 0.001**
BMI, kg/m^2^	28.5 ± 4.4	25.2 ± 3.0	26.3 ± 3.8	27.9 ± 5.0	0.055
Usage years of CPAP, year	9.0 ± 3.9	8.6 ± 2.9	8.8 ± 3.2	8.1 ± 3.9	0.573
AHI, no/h	4.8 ± 2.5	9.5 ± 7.3	7.9 ± 6.5	6.3 ± 4.8	0.210
Standard CSB%, %	4.1 ± 4.6	10.6 ± 10.2	8.4 ± 9.2	4.4 ± 6.1	**0.013**
CPAP usage time, min	389.0 ± 73.0	430.6 ± 64.7	416.7 ± 69.3	397.1 ± 80.0	0.114
CPAP usage day, %	97.9 ± 4.0	100 ± 0	99.3 ± 2.5	96.6 ± 8.4	**0.001**
**Smoking status**, ***n*** **(%)**					
Current smoker	0 (0)	0 (0)	0 (0)	18 (12.1)	**0.047**
Former smoker	7 (63.6)	21 (95.5)	27 (81.8)	75 (50.3)	***p*** **< 0.001**
Non-smoker	4 (36.4)	1 (4.5)	5 (15.2)	56 (37.6)	**0.014**
**Frequency of alcohol consumption**, ***n*** **(%)**					
≥3 times/wk	2 (18.2)	1 (4.5)	3 (9.1)	39 (26.2)	**0.040**
1–2 times/wk	0 (0)	4 (18.2)	4 (12.1)	18 (12.1)	1
<1 times/wk	2 (18.2)	3 (13.6)	5 (15.2)	13 (8.7)	0.330
Rarely or nevever	7 (63.6)	14 (63.6)	21 (63.6)	79 (53.0)	0.335
History of congestive heart failure	0 (0)	22 (100)	22 (66.7)	25 (16.8)	***p*** **< 0.001**
History of ischemic heart disease	2 (18.2)	3 (13.6)	5 (15.2)	13 (8.7)	0.330
Prior stroke/TIA	1 (9.0)	5 (22.7)	6 (18.2)	12 (8.1)	0.103
Chronic kidney disease above stage3b	3 (27.3)	6 (27.3)	9 (27.3)	12 (8.1)	**0.005**
eGFR(Cr), mL/min/1.73 m^2^	52.5 ± 12.4	50.7 ± 17.9	51.3 ± 16.9	64.2 ± 15.6	***p*** **< 0.001**
Artrial fibrillation	7 (63.6)	19 (86.4)	26 (78.8)	45 (30.2)	***p*** **< 0.001**
Hypertension	10 (90.9)	14 (63.6)	24 (72.7)	95 (63.8)	0.420
Type 2 diabetes	8 (72.7)	3 (13.6)	11 (33.3)	34 (22.8)	0.264
Dyslipidemia	6 (54.5)	11 (50.0)	17 (51.5)	64 (43.0)	0.440
Chronic obstructive pulmonary disease	1 (9.0)	3 (13.6)	4 (12.1)	47 (31.5)	**0.031**
Pacemaker implanted	1 (9.0)	2 (9.1)	3 (9.1)	1 (0.1)	**0.019**
QRS duration on ECG, ms	106.5 ± 22.6	105.5 ± 21.0	105.8 ± 21.2	99.5 ± 15.1	0.154
Central nervous system agents	2 (18.2)	0 (0)	2 (6.1)	9 (0.6)	0.358

*N (%), or mean ± SD*.

*AHF, acute heart failure; CHF, chronic heart failure; CPAP, continuous positive airway pressure; BMI, body mass index; AHI, apnea-hypopnea index; CSB, Cheyne-Stokes breathing; GFR, glemerular filtration rate; TIA, transient ischemic attack; ECG, electrocardiogram*.

*Statistical signifcance is indicated in bold*.

### Clinical Data at the Base Date of HF

Table 2 shows the clinical data at the base date of HF of 11 patients with AHF and 22 patients with exacerbated CHF. According to the Clinical Scenario classification, the AHF group consisted of four patients with CS1, five patients with CS2, one patient with CS3, and one patient with CS4. In the CHF group, according to the New York Heart Association functional classification, the patients were classified as class I or IIs during the stable period, whereas there were three patients with class IIm, 18 patients with class III, and one patient with class IV during the exacerbation period. According to classified with LVEF, most of patients were HFpEF (HFrEF; 1 case, HFmrEF; 1 case and HFpEF; 20 cases). Hypertrophic, dilated and restrictive cardiomyopathy, Takotsubo cardiomyopathy, arrhythmogenic cardiomyopathy, etc. None of these are included.

**Table 2:** Comparison of clinical data at the base date of heart failure.

**Variables**	**AHF (*n* = 11)**	**CHF (*n* = 22)**	***p-*** **value**
**Clinical symptoms**			
Dyspnea on effort	10 (91.0)	20 (91.0)	1
Orthopnea	10 (91.0)	1 (21.3)	**0.002**
Peripheral edema	9 (82.0)	21 (95.5)	0.252
Heart failure classification			
CS1/2/3/4, NYHAIIm/III/IV	4/5/1/1	3/18/1	
**Etiology of heart failure**			
Atrial fibrillation	5 (45.5)	18 (81.8)	**0.049**
Ischemia	2 (18.2)	2 (9.1)	0.586
Hypertension	2 (18.2)	0	0.104
Bradyarrhythmia	1 (9.1)	2 (9.1)	1
Valvular disease	1 (9.1)	0	0.333
Brain natriuretic peptide	301.8 ± 157.5	275.1 ± 205.2	0.851
**Echocardiogram findings**			
Left ventricular ejection fraction	60.7 ± 10.2	61.3 ± 9.6	0.851
Left ventricular end-diastolic diameter, mm	52.7 ± 5.5	56.0 ± 6.1	0.098
Left ventricular end-systolic diameter, mm	35.7 ± 7.3	36.7 ± 5.7	0.277
Left atrial dimension, mm	48.6 ± 5.7	56.0 ± 13.8	0.138
Transtricuspid pressure gradient, mmHg	34.8 ± 13.1	37.5 ± 13.3	0.720
Mitral valveregurgitation I/II/III/IV	4/5/1/1	2/11/2/1	
E/e'	15.8 ± 6.2	15.7 ± 4.8	0.965
**Medications**			
ACE inhibitors and/ ARBs	2/8 (91.0)	0/15 (68.2)	0.069
β-blockers	2 (18.2)	16 (72.7)	**0.008**
Mineralocorticoid receptor antagonist	0	7 (31.8)	0.067
Diuretics	0	5 (22.7)	0.143
Digitalis	1 (0.9)	0	0.333
Nitrate	0	4 (18.2)	0.276

*N (%), or mean ± SD*.

*AHF, acute heart failure; CHF, chronic heart failure; CS, clinical scenario; NYHA, New York Heart Association functional classification; E/e', ratio of early diastolic mitral inflow velocity to septal mitral annulus tissue relaxation velocity; ACE, angiotensin converting enzyme; ARB, angiotensin II receptor blocker. Echocardiogram: Values missing for 6 patients with CHF*.

*Statistical signifcance is indicated in bold*.

In the CHF group, exacerbation appeared to be attributable to Af in significantly more patients during the exacerbation period (81.8 vs. 45.5%, *P* < 0.049). Echocardiogram findings showed that the left ventricular ejection fraction was preserved in many patients in both groups (60.7 ± 10.2 vs. 61.3 ± 9.6, *P* = 0.851), and that the transtricuspid pressure gradient (34.8 ± 13.1 vs. 37.5 ± 13.3, *P* = 0.720) and ratio of early diastolic mitral inflow velocity to septal mitral annulus tissue relaxation velocity (E/e') (15.8 ± 6.2 vs. 15.7 ± 4.8, *P* = 0.965) tended to be higher.

### Comparisons of Chronological Variations in CSB% Among the AHF, CHF, and Control Groups

**Figure 4:**
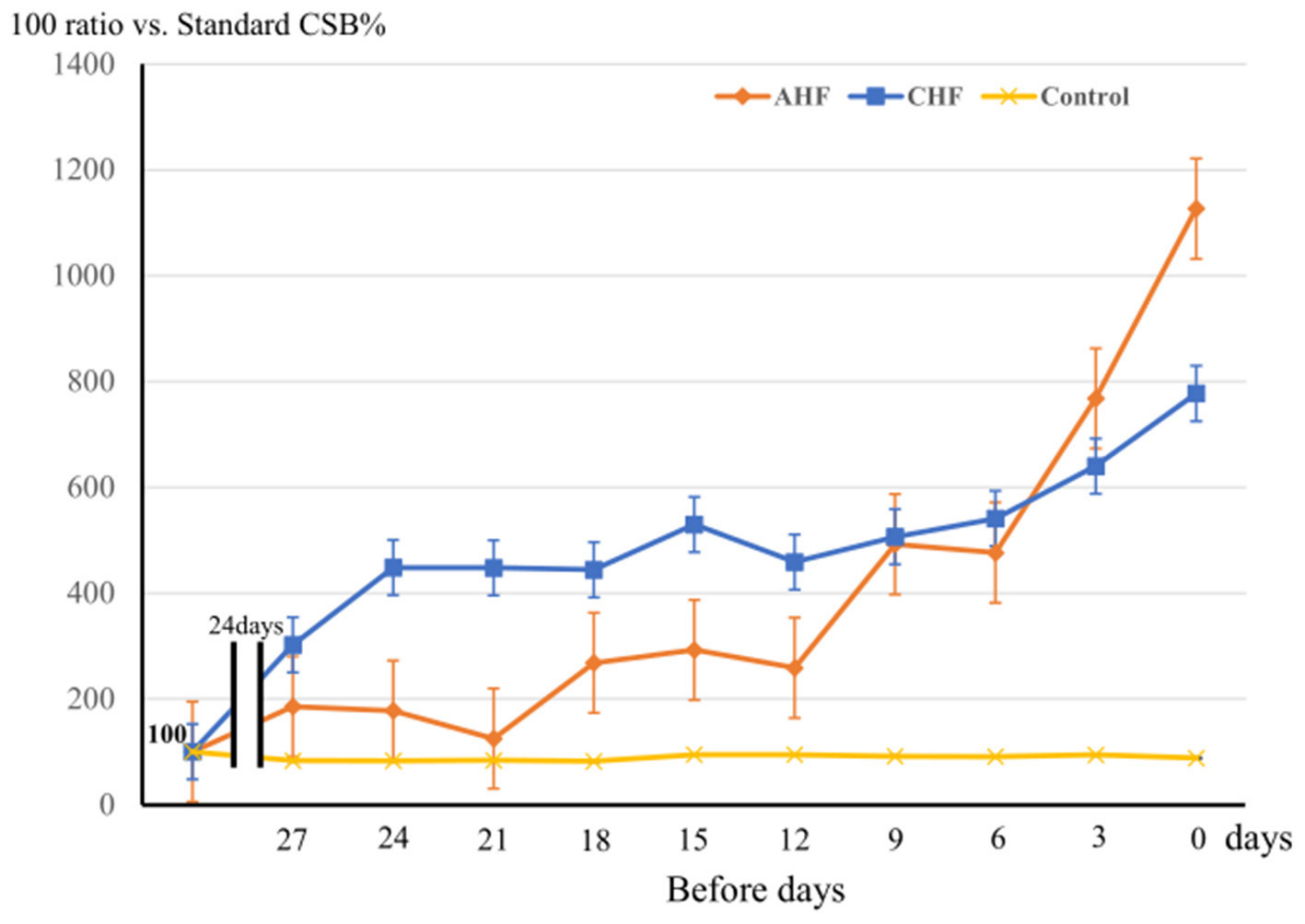
The variation of CSB% among 3 groups during the 30 days prior to the base date is shown. Three groups: the acute heart failure (AHF) group (*n* = 11), chronic heart failure (CHF) group (*n* = 22), and control group (*n* = 149). The standard CSB% was defined as 100. The 30-day period to the base date was divided into 10 3-day periods, and the average CSB% was calculated as a ratio to the standard CSB% (100) for each period. Error bars indicate the standard error. Analyzed them with a two-way repeated measures ANOVA. Factor.Group, *p* < 0.001, Factor.Group:Time, *p* < 0.001.

Figure 4 shows the variations in the average CSB% in the three groups comprising the AHF group (11 patients), CHF group (22 patients), and control group (149 patients). The standard CSB% was defined as 100. The 30 days prior to the base date were divided into 10 periods every 3 days, and the average CSB% for each 3 days was calculated and plotted as a ratio of the standard CSB% of 100. There was a significant difference in CSB% among the three groups (Factor.Group, *p* < 0.001). Furthermore, the chronological change in CSB% among the three groups was significantly different (Factor.Group:Time, *p* < 0.001).

In the control group, the CSB% did not vary markedly over the 30-days period, whereas the CSB% in the CHF group gradually increased and remained high until the day of admission or hospital visit. In the AHF group, the CSB% started linear increasing rapidly ~10 days before admission.

### Comparisons of SD CSB% Over a 30-Days Period Among the AHF, CHF, and Control Groups

**Figure 5:**
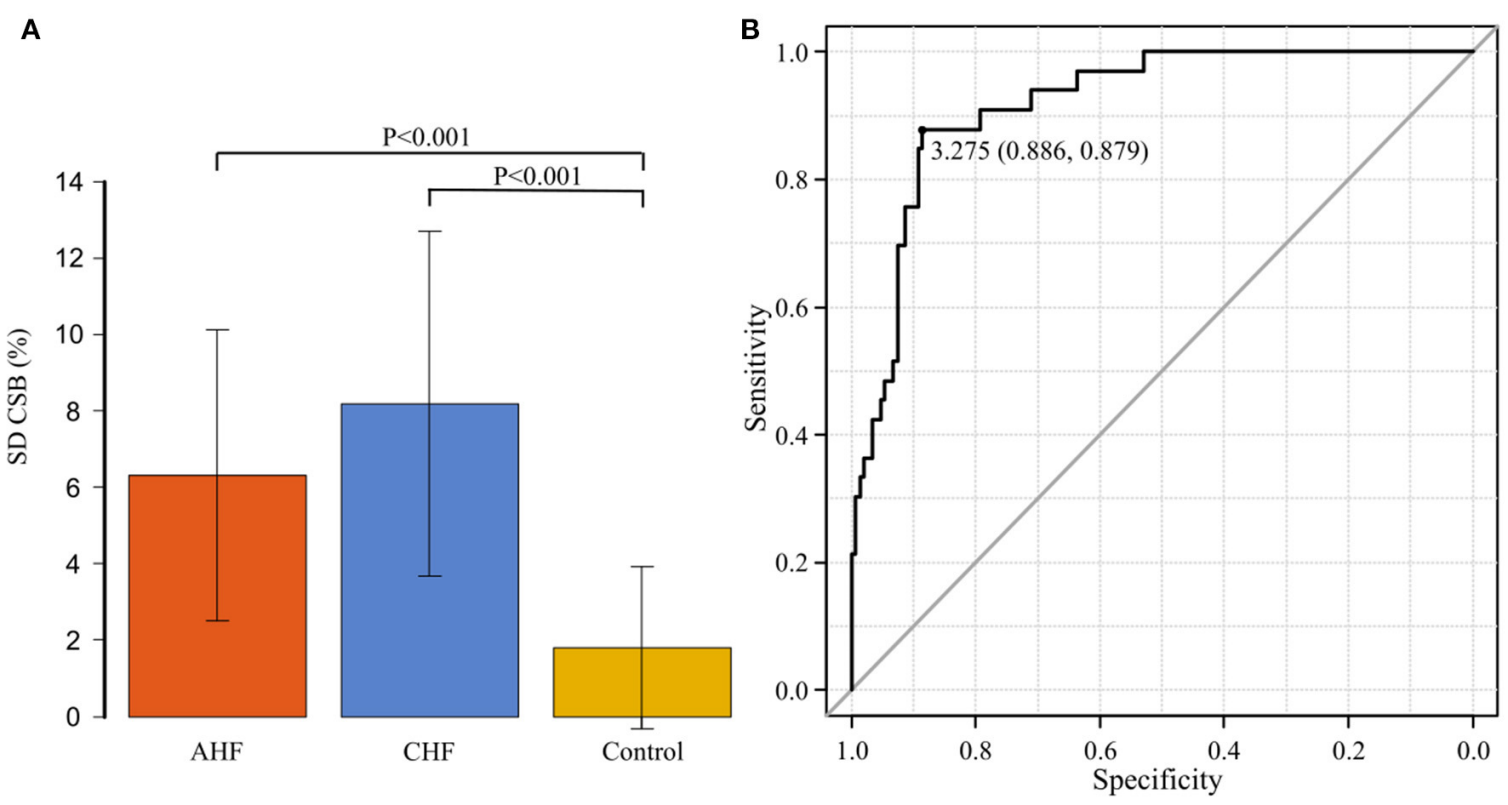
**(A)** The standard deviation of the Cheyne-Stokes breathing rate (SD CSB%) is compared among the acute heart failure (AHF) group (*n* = 11), chronic heart failure (CHF) group (*n* = 22), and control groups (*n* = 149). The average SD CSB% was significantly higher in the AHF and CHF groups than in the control group (*P* < 0.001 and *P* < 0.001, respectively). **(B)** Receiver operating characteristic curve was generated with the presence or absence of the onset of heart failure as the dependent variable to determine the cut-off value of the standard deviation of the Cheyne-Stokes breathing rate. The area under the curve was 0.919 (95% confidence interval: 0.874–0.964, *P* < 0.001). The cut-off point was 3.275 (specificity of 0.886 and sensitivity of 0.879).

Figure 5A shows the comparison of SD CSB% among the AHF group (11 patients), CHF group (22 patients), and control group (149 patients). The average SD CSB% was 6.3 ± 3.8 in the AHF group, 8.2 ± 4.6 in the CHF group, and 1.8 ± 2.1 in the control group. The CHF group had the highest value. Compared to the average SD CSB% in the control group, average SD CSB% in the AHF and CHF groups was significantly higher (*P* < 0.001 and *P* < 0.001, respectively).

### ROC Curve for SD CSB% at the Onset of HF

An ROC curve was generated with the presence or absence of the onset of HF as the dependent variable to determine the cut-off value of SD CSB% (Figure 5B). The area under the curve (AUC) was 0.919 [95% confidence interval (CI): 0.874–0.964, *P* < 0.001], indicating a high accuracy. The cut-off point was 3.275 (specificity of 0.886 and sensitivity of 0.879).

### Comparison of CL Between 22 CHF Patients and 44 Pair Matched Controls

Table 3 shows the comparison of baseline characteristics and clinical data between 22 patients in the CHF group and 44 patients who were pair marched for the three variables of sex, BMI, and standard CSB% in a ratio of 1: 2 from the 149 patients in the control group. All these patients were men. In the CHF group, the comorbidity rate of Af (86.4 vs. 36.4%, *P* < 0.001), and the proportion of patients treated with two or more types of antihypertensive drugs (90.9 vs. 40.9%, *P* < 0.001) were significantly higher than in the control group. The echocardiographic findings showed no significant difference in the left ventricular systolic performance (61.3 ± 9.6% vs. 65.4 ± 5.5%, *P* = 0.051). The length of the left atrial dimension (56.0 ± 13.8 mm vs. 40.5 ± 6.7 mm, *P* < 0.001), transtricuspid pressure gradient (37.5 ± 13.3 mmHg vs. 25.7 ± 7.8 mmHg, *P* = 0.031), and E/e' (15.7 ± 4.8 vs. 10.1 ± 3.0, *P* < 0.001) were significantly higher in the CHF group. In the CHF group, 15 of 22 patients had an H2FPEF score of 6 or higher (12).

**Table 3:** Comparison of Baseline characteristics and CPAP Device data of chronic heart failure group vs. pair matched control group.

**Variables**	**CHF (*n* = 22)**	**Control (*n* = 44)**	***p-*** **value**
Male (%)	22 (100)	44 (100)	1
Age, year	77.0 ± 4.2	71.4 ± 10.7	**0.011**
BMI, kg/m^2^	25.2 ± 3.0	25.3 ± 3.1	0.693
Standard CSB% (month), %	10.6 ± 10.2	6.0 ± 6.0	0.090
Cycle length (day), ms	79.3 ±11.9	59.0 ± 7.5	**<0.001**
Cycle length during exacerbations (day), ms	85.1 ±11.5		^*^0.004
History of congestive heart failure	22 (100)	2 (4.5)	**<0.001**
History of ischemic heart disease	3 (13.6)	5 (11.4)	1
Prior stroke/TIA	5 (22.7)	1 (2.3)	**0.014**
Artrial fibrillation	19 (86.4)	16 (36.4)	**<0.001**
Hypertension	14 (63.6)	33 (75.0)	0.393
Pacemaker implanted	2 (9.1)	1 (2.2)	0.256
QRS duration on electrocardiogram, ms	105.5 ± 21.0	101.0 ± 19.0	0.447
2 or more antihypertensive medicines	20 (90.9)	18 (40.9)	**<0.001**
central nervous system agents	0 (0)	1 (2.3)	1
^**^Echocardiogram findings			
Left ventricular ejection fraction	61.3 ± 9.6	65.4 ± 5.5	0.051
Left atrial dimension, mm	56.0 ± 13.8	40.5 ± 6.7	**<0.001**
Transtricuspid pressure gradient, mmHg	37.5 ± 13.3	25.7 ± 7.8	**0.031**
E/e'	15.7 ± 4.8	10.1 ± 3.0	**0.001**
H2EPEF score > 6	15 (68.2)	3 (6.8)	**<0.001**

*N (%), or mean ± SD*.

*CPAP, continuous positive airway pressure; CHF, chronic heart failure; BMI, body mass index; CSB, Cheyne-Stokes breathing; TIA, transient ischemic attack; E/e', ratio of early diastolic mitral inflow velocity to septal mitral annulus tissue relaxation velocity*.

* *Wilcoxon signed rank test with continuity correction*.

** *Echocardiographic findings in the CHF group were measured during exacerbations*.

*Echocardiogram: Values missing for 6 patients with CHF*.

*Echocardiogram: Values missing for 13 patients with Control*.

*Statistical signifcance is indicated in bold*.

**Figure 6:**
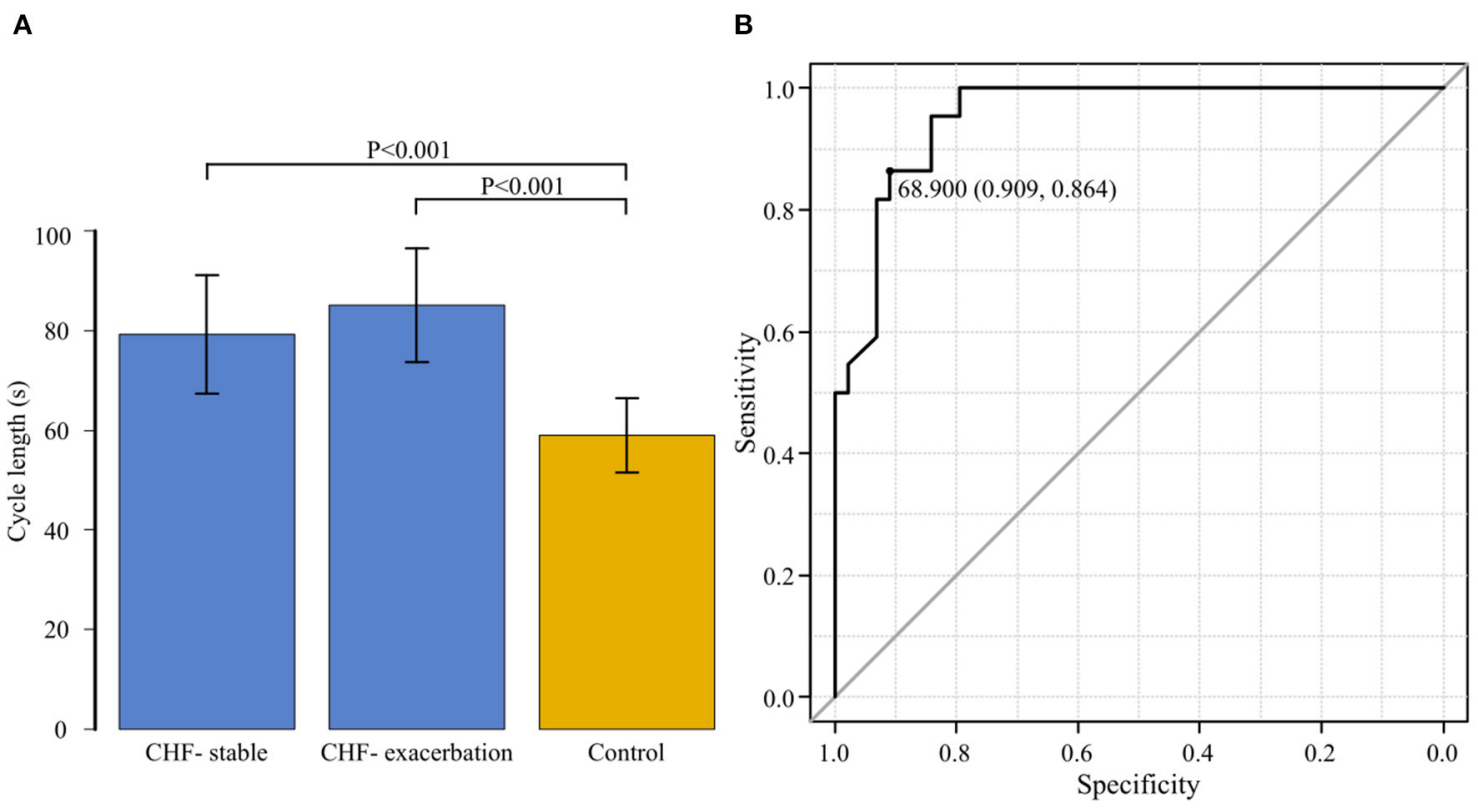
**(A)** The cycle length is compared among the three groups: chronic heart failure (CHF) during the stable period (CHF-stable), CHF during the exacerbation period (CHF-exacerbation), and controls. Compared to the average cycle length in the control group (59.5 ± 9.1 s), that in both the CHF-stable and CHF-exacerbation groups was significantly longer (*P* < 0.001 and *P* < 0.001, respectively). **(B)** A receiver operating characteristic curve for the group of patients with chronic heart failure (CHF) during the stable period and the control group was generated with the presence or absence of exacerbation of CHF as the dependent variable to determine the cut-off value of the cycle length. The area under the curve was 0.954 (95% confidence interval: 0.910–0.997, *P* < 0.001). The cut-off point was 68.9 s (specificity of 0.909 and sensitivity of 0.864).

The average CL in the CHF group was 79.3 ± 11.9 s during the stable period and 85.1±11.5 s during the exacerbation period. It was significantly longer during the exacerbation period (*P* = 0.004). Figure 6A shows the comparison of CL among the three groups comprising the CHF-stable, CHF-exacerbation, and control groups. Compared to the average CL in the control group (59.5 ± 9.1 s), average CL in both the CHF-stable and CHF-exacerbation groups was significantly longer (*P* < 0.001 and *P* < 0.001, respectively).

### ROC Curve for CL During the Stable Period in the CHF and Control Groups

While the CHF-exacerbation group was regarded as the HF group, an ROC curve for the CHF-stable and control groups was generated with the HF group as the dependent variable to determine the cut-off value of CL of CSB (Figure 6B). The AUC was 0.954 (95% CI: 0.910–0.997, *P* < 0.001), indicating a high accuracy. The cut-off point was 68.9 s (specificity of 0.909 and sensitivity of 0.864).

## Discussion

Based on the CPAP remote monitoring data, we previously examined the percentage of CSB duration during sleep (CSB%) in 618 patients with stable OSA who were on CPAP for extended periods (13). The median CSB% was as low as 0.32%, while 149 patients (24.1%) had a CSB% of 1% or higher. Perpetuate of Af and an increase in the QRS duration were found to be the major predictive indicators. In the present study, the 149 patients with a CSB% of 1% or higher who were examined in our previous study were used as the control group and compared for variations in the CSB% and CL of CSB with 33 patients who actually developed HF. The CSB% began increasing ~10 days before the onset in the AHF group, and ~1 month before the onset in the CHF group. The SD CSB% was significantly higher in both the AHF and CHF groups, compared to the control group. The cut-off SD CSB% that could predict the onset of HF was 3.275, with a favorable sensitivity and specificity of 0.886 and 0.879, respectively. In addition, the CL of CSB was significantly longer in the CHF group during both the stable and exacerbation periods than in the control group. Even in the same patient in the CHF group, the CL was significantly longer during the exacerbation period than during the stable period. The cut-off value of CL that could predict the onset of HF was 68.9 s, with a favorable sensitivity of 0.909 and specificity of 0.864.

### Linkage Between Cardiovascular Dynamics and Variability of CSB

Most previous studies on the association between CSA-CSB and HF have analyzed polysomnography data collected at the time of diagnosis from patients with HFrEF, instead of those with HFpEF. Important pathophysiological factors for CSB are the responses of various receptors involved in respiratory control. Particularly in patients with HF, CSB is considered to be affected by increased left atrial pressure, pulmonary congestion, circulatory delay, and enhanced sympathetic nervous activity (14). Cardiac output decreases, and pulmonary vascular pressure increases. Increased pulmonary vascular pressure stimulates the pulmonary vagal afferent receptors to cause hyperventilation and hypopnea (15, 16). These CSA-CSB-associated events have been considered to be inversely associated with the cardiac output (17).

On the other hand, Bitter et al. have reported that the prevalence of CSB is high in Af patients even with normal systolic left ventricular function (18). In other words, they have discussed that CSB is more likely to be attributable to left ventricular end diastolic pressure or pulmonary congestion than to systolic left ventricular function itself.

Regarding HFpEF, Bitter et al. also reported that adaptive servo-ventilation (ASV) improved left ventricular diastolic function and exercise capacity of HFpEF patients with CSB (19). Yoshihisa et al. claimed that ASV reduced rehospitalization and/or cardiac mortality of HFpEF patients with sleep disordered breathing with favorable effects such as improvement of left ventricular diastolic function and arterial stiffness (20). Moreover, they reported that timely application of CPAP and ASV improved the respiratory function, right-heart function, exercise tolerance, and prognosis in patients with HFpEF complicated by SAS (21).

In addition, D'Elia et al. reported that the acute use of ASV seems effective in reducing brain natriuretic peptide and improving diastolic and right ventricular function in acute HFpEF patients with sleep disordered breathing SDB and CSA (22). According to them, it is particularly important to consider the associations of diastolic left ventricular function, right-heart function, and exercise tolerance with CSB when the prognosis of HF is being evaluated.

In our previous study based on the monitoring data from the CPAP device, 83.3% of the emergence group, which included patients whose CSB% was <1% at the time of diagnosis but subsequently increased to 2% or higher over time, experienced at least one of the following events during the period from polysomnography at the time of diagnosis to analyses of the CPAP device data: new onset or relapse of HF, cerebral infarction, and Af, as well as diastolic dysfunction and increased QRS duration. Left ventricular ejection fraction was 45% or higher in all patients (13).

Based on these results, we assume that the increase in CSB% over time reflects the new onset of cerebrovascular and cardiovascular events, increased left atrial pressure, and exacerbation of diastolic dysfunction and pulmonary congestion rather than decreased left ventricular ejection fraction. This suggests that monitoring of exacerbation of left atrial pressure and pulmonary congestion through chronological variations in CSB% may contribute to early detection of the onset and exacerbation of HF.

In the present study, the optimal cut-off value of SD CSB that could predict the onset of HF was 3.275. However, because the CSB% in the early periods was high in patients with persistent AF and other conditions, it was difficult to determine the cut-off point based only on the increase and variations in CSB in some patients.

### Linkage Between Cardiovascular Dynamics and Cycle Length of CSB

Regarding the CL of CSB, Wedewardt et al. reported that, even among patients with similar AHI, the CL was longer in those with severe HF (23). Javed et al. demonstrated in the SERVE-HF substudy analysis that cardiovascular mortality and rate of hospitalization due to exacerbation of HF were higher in patients with HFrEF and longer CSB, and that the median CL in these patients was 64.5 and 62.1 s, respectively (24). Both reports showed a correlation between decreased cardiac output and increased CL of CSB.

In addition, Giannoni et al. showed that the lung-to-finger circulation time was the only independent predictive indicator of CL of CSB (25). Spiesshoefer et al. reported that a significant improvement in the left atrial pressure and circulatory delay, as well as a significant reduction in the ventilation length during central sleep hypopnea, was observed within 5 days after MitraClip implantation (26).

These reports also suggest that the CL of CSB reflects increased left atrial pressure and circulatory delay.

In the present study, which predominantly included patients with HFpEF, the CL was significantly increased during exacerbation of CHF even in the same patient. When we compared the CL between the CHF-stable and control groups, the CL in the CHF-stable group was significantly longer. Furthermore, patients with a short CL did not develop HF, despite a high CSB%. The optimal cut-off point that could predict the onset of HF was 68.9 s.

Consequently, we think that monitoring the CL over time may contribute to prediction of HF exacerbation, stratification of CSA-CSB cases that should be treated, and development of criteria for switching from CPAP to ASV.

### Limitations

The present study has the following limitations.

First, the number of patients with heart failure was small (33 patients), and most of the patients with CHF exacerbation were elderly men.
Second, the HF patients were skewed toward HFpEF with AF rather than typical HFrEF.
The third point is that the analysis algorithm from CPAP machine is not standardized.
Therefore, the standard values of SDCSB% and CL in this study cannot be generalized as they are.
However, we believe that we were able to suggest the possibility of early prediction of the onset and exacerbation of HF by monitoring changes in CSB% and CL.

We believe that this is an event worthy of further investigation in a prospective, multicenter study including HFrEF or women.

Furthermore, in order to improve the accuracy of remote monitoring, it is desirable to improve the reliability and performance of analysis software, and to establish automated measurement of the CL of CSB.

Monitoring of CSB% alone is insufficient for making decisions about the treatment of patients with Strotmann et al. reported that CSB was present in 34% of patients with AF and Preserved ejection fraction (27). How can the exacerbation of concurrent persistent Af be differentiated? Based on the results of the present study, addition of CL to monitoring may be expected to further improve the accuracy of detecting abnormalities.

## Conclusion

Using CPAP remote monitoring data of patients with OSA on CPAP, we retrospectively compared variations in CSB% and CL between the HF and control groups.

The CSB% showed greater variations in the HF group than in the control group. The CSB% started increasing ~10 days before the onset in the AHF group and ~1 month before the onset in the CHF-exacerbation group. The CL of CSB was significantly longer in the HF group than in the control group, and was longer during the exacerbation period than during the stable period.

The present study suggests that chronologically monitoring of the incidence and CL of CSB with a CPAP device might allow early prediction of the onset of HF.

## Data Availability Statement

The raw data supporting the conclusions of this article will be made available by the authors, without undue reservation.

## Ethics Statement

The studies involving human participants were reviewed and approved by Institutional Review Board (IRB) of Medical Corporation MSC (#20002, UMIN00000042555). The patients/participants provided their written informed consent to participate in this study.

## Author Contributions

KS designed the study and wrote the initial draft of the manuscript. YT contributed to data collection, analysis, and interpretation of data. All authors contributed to the article and approved the submitted version.

## Conflict of Interest

The authors declare that the research was conducted in the absence of any commercial or financial relationships that could be construed as a potential conflict of interest.

## Publisher's Note

All claims expressed in this article are solely those of the authors and do not necessarily represent those of their affiliated organizations, or those of the publisher, the editors and the reviewers. Any product that may be evaluated in this article, or claim that may be made by its manufacturer, is not guaranteed or endorsed by the publisher.

## Abbreviations

HF: heart failure
OSA: obstructive sleep apnea
CSA: central sleep apnea
CSB: Cheyne-Stokes breathing
ASV: adaptive servo ventilation
CPAP: continuous positive airway pressure
AHF: acute heart failure
CHF: chronic heart failure
LVEF: left ventricular ejection fraction
HFrEF: HF with reduced EF
HFmrEF: HF with mid-range EF
HFpEF: HF with preserved EF
AHI: apnea-hypopnea index
Af: atrial fibrillation
ROC: receiver operating characteristic curve
BMI: body mass index
CL: cycle length
AUC: area under the curve
SD: standard deviation.
